# Triangular Silver Nanoplates as a Bioanalytical Tool: Potential COVID-19 Detection

**DOI:** 10.3390/ijms241511974

**Published:** 2023-07-26

**Authors:** Laura G. Rodriguez Barroso, Eduardo Lanzagorta Garcia, Marija Mojicevic, Buket Alkan Tas, Miriam Huerta, Robert Pogue, Declan M. Devine, Margaret Brennan-Fournet

**Affiliations:** 1 PRISM Research Institute, Technological University of the Shannon: Midlands Midwest, Dublin Rd, N37 HD68 Athlone, Ireland; laura.rodriguez@tus.ie (L.G.R.B.); e.lgarcia@research.ait.ie (E.L.G.); buket.tas@tus.ie (B.A.T.); declan.devine@tus.ie (D.M.D.); margaret.brennanfournet@tus.ie (M.B.-F.); 2 Physics Institute, Universidad Autónoma de San Luis Potosí, Av. Parque Chapultepec 1570, San Luis Potosí 78295, Mexico; miriam.huerta@if.uaslp.mx; 3 Campus Asa Norte, Universidade Católica de Brasília, SGAN Módulo B 916 Avenida W5, Brasilia 70790-160, Brazil; redward@p.ucb.br

**Keywords:** TSNP, nanoplates, fibronectin, immunoassay, COVID-19, spike, immunodetection

## Abstract

Nanotechnology offers new possibilities in molecular diagnostics, with nanoparticles gaining attention as biosensor upgrades. This study evaluates gold-coated silver nanoplates coated with PEG for enhanced protection, aiming to detect Spike protein with higher sensitivity, and emphasizes the importance of considering complex environments and appropriate controls for specific binding and accurate analysis. The sensitivity of antibody-coated PEGAuTSNPs as tools for immunoassays is demonstrated through fibronectin (Fn)– anti-fibronectin binding within an isolated extracellular matrix as a complex and native environment of Fn. Moreover, the optimal functionalization volume of Spike protein was determined (4 µg/mL of PEGAuTSNP). Anti-Spike was added to confirm binding, while the TJP1 protein was used as a negative control. The same experiment was used in the presence of horse serum to simulate a complex environment. According to Localized Surface Plasmon Resonance analysis and Dynamic Light Scattering size measurements, anti-Spike exhibited a stronger affinity for the nanoplates, causing TJP1 to be replaced by the antibody on the nanoplates’ surface. Future research will involve exploring alternative complex environments, filtering larger molecules, and the optimization of immunoassay performance.

## 1. Introduction

Immunoassays (IAs) have been used for decades for various bioanalytical applications, ranging from clinical diagnostics and pharmaceutical analyses to environmental and food monitoring and testing. The early-stage and precise detection of analytes has been recognized as a crucial factor in these applications [1]. Successful monitoring, disease prevention and overall care for public health rely on the effectiveness of biomarkers regarding food and clinical samples. IAs are based on the quantitation of an analyte through the reaction of an antigen (Ag) and an antibody (Ab) [2,3]. They are selective and sensitive; however, labeling agents are commonly used to increase sensitivity and catalyze the reaction [4]. Moreover, these methods involve laborious protocols and, in some cases, take several days to complete a reaction [5]. Similar limitations are present in enzyme-linked immunosorbent assays (ELISAs), one of the commonly used IA procedures based on the catalytic potential and specificity of enzymes to detect antigen–antibody reactions. Additionally, the application of enzymes is usually accompanied by low stability and non-specificity, together with large amounts of the required sample to perform the test [6,7]. Antibodies, a family of related glycoproteins, are being extensively used as the biorecognition element in various immunoassays, while their utilization in the field of biosensors offers potential for the development of new bioanalytical tools. IAs’ selectivity and high sensitivity toward analytes ensure specific interactions, resulting in highly effective and reliable test results [8].

The currently available techniques for protein characterization that are able to detect conformations and structural changes in proteins require expensive instrumentation and extensive data analysis, and their signals are obstructed by cellular noise when applied in complex biological environments [9]. The necessity for the development of enzyme-free analytical assays has arisen in order to overcome ELISA assays’ limitations [10]. Thus, the accurate and rapid detection of newly emerging diseases is crucial to prevent transmission and allow rapid treatment for affected individuals. For instance, the COVID-19 pandemic has had a significant impact in the world, resulting in the loss of millions of lives. It has emerged as one of the most consequential global health crises since the influenza pandemic [11], leading to the development and commercialization of a significant number of ELISAs that allow the detection of antibodies binding to structural proteins [12,13]. However, the emergence of nanotechnology has created new possibilities when it comes to molecular diagnostics. 

The utilization of nanoparticles has become an attractive option for upgrading biosensors; because of their small size, a greater surface to volume ratio is provided, accompanied with their inherent physicochemical properties and decreased sensitivity to external factors [14]. Noble metal nanoparticles are known to have remarkable optical, catalytic, electronic and magnetic properties and have been intensely researched for the development of highly sensitive nano-biosensors to investigate a range of molecules and detect specific biomarkers [15,16]. Recent research has presented metallic nanoparticles as a promising tool for the detection of COVID-19, establishing new research pathways for the development of a range of techniques in assisting the prompt response to rising disease challenges. To date, evidence indicates that SARS-CoV-2 targets various organs, including the kidneys, heart, and lungs, leading to the eventual failure of multiple organ systems; hence, the necessity of developing accurate systems for its detection is crucial [17]. Silver nanoparticles have been used to improve glucose and hydrogen peroxide biosensors [18,19]. Moreover, they have been utilized for the dramatic enhancement of electrochemical IAs by coupling nanoparticles with enzyme catalytic effects [20]. It was reported that silver nanoparticles used as electrochemical labels have significantly improved IAs for the detection of tick-borne encephalitis virus Ab [21]. Specifically, triangular silver nanoparticles (TSNPs) have appealing tunable plasmonic characteristics and have been used for biosensing applications. Furthermore, their optical profile has been reported to show the strongest and sharpest peaks among other metals [22]. Moreover, gold-edge-coated triangular silver nanoparticles (AuTSNPs) with enhanced sensitivity have proven to be powerful new tools to monitor protein activity. This versatile method has the potential to provide the accurate and rapid detection of viral proteins and lead to better treatments for emerging infections and global outbreaks [23]. 

In this study, the high sensitivity of AuTSNPs as a tool for immunoassays is demonstrated through an analysis of Ab-Ag binding within an isolated extracellular matrix (ECM) as a complex environment, where anti-fibronectin (Fn) functionalized nanoplates bind to the corresponding antigen in its native environment. Moreover, the development of a potential platform for SARS-CoV-2 Spike protein detection is presented. This technique holds promise for adaptation and application in future outbreaks. 

## 2. Results

### 2.1. Detection of Native Fn Using Anti-Fn Antibody-Functionalized PEGAuTSNP

**Figure 1:**
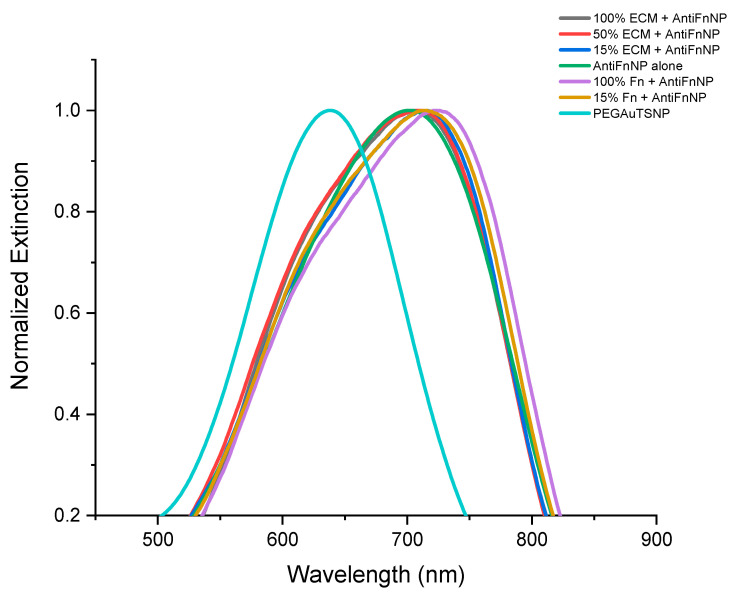
LSPR spectra of anti-Fn-native Fn binding experiment.

The sensitivity of Fn antibody-functionalized nanoparticles to detect their corresponding antigen within its native environment was tested. Anti-Fn-PEGAuTSNPs were exposed to different concentrations of cell-extracted extracellular matrix (ECM) as a complex and native environment for Fn. As presented in Figure 1 and Table 1, according to Localized Surface Plasmon Resonance analysis (LSPR), PEGAuTSNPs’ λ_max_ was recorded at 638 nm, and upon functionalization with anti-Fn, a shift of 65 nm was observed, indicating the successful coating of the nanoplates with the antibody. Following this, the functionalized nanoplates were exposed to different concentrations of cell-isolated ECM and analyzed, showing a shifting profile, different than expected, with longer shifts recorded for the less concentrated ECM samples.

**Figure 2:**
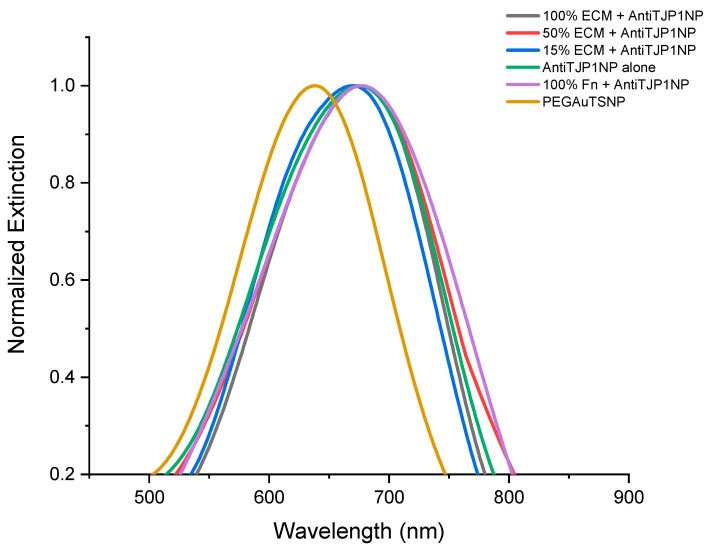
LSPR spectra of anti-TJP1-Anti-Fn binding experiment.

For all concentrations of ECM, the shift recorded was positive, from 4 nm to 12 nm shifts indicating the binding of anti-Fn-PEGAuTSNP to native Fn. This was validated with the addition of 100% and 15% purified Fn (non-native) as positive controls, where expected redshifts of 20 nm and 11 nm, respectively, were recorded (Figure 1). Further testing was carried out with the use of anti-TJP1-PEGAuTSNP as a negative control for binding, since this human tight junction protein 1 (TJP1)-targeted antibody should not show specificity to Fn. As shown in Figure 2 and Table 2, a shift of 37 nm was recorded for PEGAuTSNPs upon the addition of anti-TJP1, indicating the functionalization of the nanoparticles with the antibody. When the nanoparticles were exposed to the different concentrations of ECM extract, limited shifts between 1 and 2 nm were observed. Similar behavior was observed when the TJP1 antibody-functionalized nanoplates were exposed to purified Fn with a 3 nm shift compared to ~20 nm shifts when Fn was exposed to anti-Fn-PEGAuTSNPs.

### 2.2. Spike Protein Optimal Functionalization Volume Determination

To determine the amount of Spike protein required to successfully cover the nanoplates’ surfaces, a range of protein concentrations were added to 1 mL of PEGAuTSNP samples and analyzed. One µg of the Spike’s corresponding antibody was added afterwards to analyze changes in the LSPR recordings. As shown in Table 3, the recorded λ_max_ for the Spike-functionalized PEGAuTSNPs was higher as the amount of Spike protein increased. Nonetheless, when between 5 µg and 10 µg concentrations of Spike were used to functionalize the nanoplates, the recorded λ_max_ started to decrease and stabilized around 632 nm, presumably indicating saturation of the nanoparticles’ surface. This was tested by adding 1 µg of the anti-Spike antibody, where a short increase in the λ_max_ was predicted as a result of the further coverage of uncoated sections of the nanoplates and the binding of the Ab-Ag complex. Upon exposure to the antibody, the results demonstrated higher λ_max_ recordings for all samples as expected; however, the λ_max_ for the samples with higher protein levels (between 5 and 10 µg) showed decreasing values, similar to the recordings prior to anti-Spike addition.

The results suggested that the protein concentration that successfully covers the nanoplates surface before the saturation point is between 3 µg and 5 µg. Considering this, a concentration of 4 µg/ mL of PEGAuTSNPs was used for further testing.

### 2.3. Spike-PEGAuTSNP Detection Limit Determination

**Figure 3:**
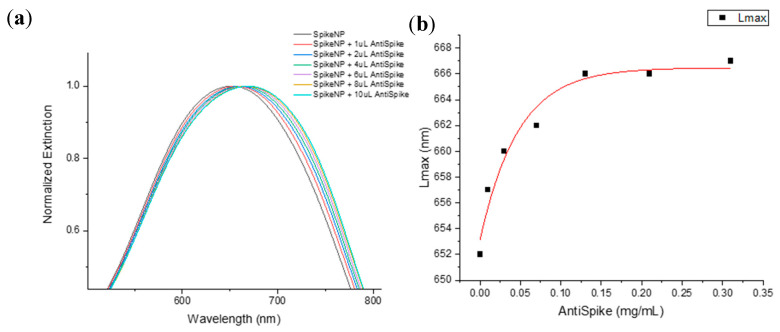
(**a**) LSPR spectra of Spike PEG-AuTSNP with increasing amounts of anti-Spike; (**b**) LSPR peak shift as a function of anti-Spike concentration for Spike PEG-AuTSNPs.

Spike-functionalized PEGAuTSNPs were used with different amounts of anti-Spike. As shown in Figure 3a, expected redshifts were recorded for increasing concentrations of anti-Spike, which suggests the intensifying binding of the antibody to the Spike-functionalized nanoplates. The binding of the Spike–anti-Spike complex could be detected in a linear range of anti-Spike concentration between 0.01 and 0.1 mg/mL (Figure 3b). Moreover, shorter redshifts were observed for the highest concentrations of antibody, indicating Ab-Ag system saturation from 0.13 mg/mL of anti-Spike. 

### 2.4. Specificity Analysis of the Spike–Anti-Spike System

**Figure 4:**
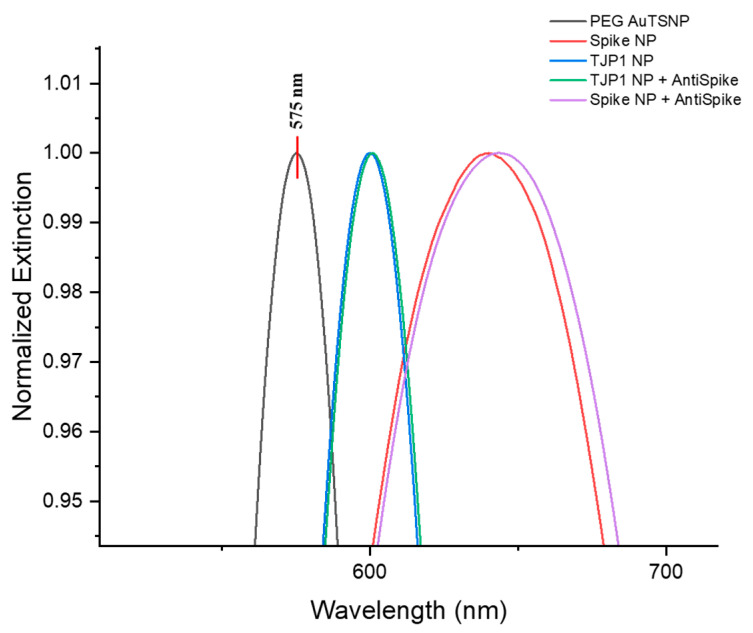
LSPR spectral shifts of PEGAuTSNPs, Spike-functionalized PEGAuTSNPs, TJP1-functionalized PEGAuTSNPs before and after exposure to anti-Spike.

The specificity of the Spike–anti-Spike system was tested with the previously established concentrations of Spike-PEGAuTSNPs and anti-Spike as per detection curves (Figure 3). The TJP1 protein was used as a negative control for binding since the anti-Spike antibody is specific to Spike protein according to the manufacturer’s information [24]. The amount of used TJP1 was equivalent to the Spike concentration to secure proper control conditions. As shown in Figure 4, the LSPR for bare PEGAuTSNPs was recorded at 575 nm, and redshifts of 65 nm (to 639 nm) and 25 nm (to 600 nm) were observed upon functionalization with Spike and TJP1 proteins, respectively. According to Table 4, a shift of 7 nm for the Spike PEGAuTSNPs was measured after exposure to its corresponding antibody, while a shift of only 1 nm was detected for the TJP1-PEGAuTSNPs.

### 2.5. Spike Protein Detection-Platform Testing within a Complex System

The binding of Spike to anti-Spike was analyzed in the presence of horse serum (HS), simulating a complex environment to study the nanoparticles’ sensitivity. TJP1 was used as a control for binding. As shown in Table 5, the λ_max_ for the Spike-functionalized PEG-AuTSNPs was initially recorded at 590 nm. As predicted, upon binding of the antibody to the Spike, a shift of 13 nm was detected. There was similar behavior in the cases where the Spike-PEGAuTSNPs were in the presence of three different concentrations of HS (100%, 50% and 10%), where positive shifts of 8 nm, 7 nm and 2 nm were recorded, respectively. When TJP1-PEGAuTSNPs (initial λ_max_ of 596 nm) were exposed to anti-Spike, a minimal shift (1 nm) was recorded, similar to previous experiments. Nonetheless, shifts of 8 nm, 3nm and 2 nm were recorded for TJP1-NP upon the addition of anti-Spike in the presence of HS.

Additional experiments were carried out, analyzing only TJP1 in order to confirm the previous results and further investigate the behavior patterns of the protein when exposed to anti-Spike in the presence of serum. PEGAuTSNPs’ λ_max_ was recorded at 589 nm, and shifts of 15 nm and 8 nm were recorded upon functionalization with Spike and TJP1, respectively. Similar to previous tests, there was a weaker attachment of TJP1 onto the nanoplates. According to Table 6, registered λ_max_ values appear to be higher upon exposure to anti-Spike in all treatments, comparable to the previous experiment. 

Moreover, shifts of 5 nm and 2 nm were recorded for TJP1-NP upon the addition of anti-Spike in the presence of HS in concentrations of 100%, 50% and 10%. As mentioned earlier, the binding of anti-Spike to the TJP1 protein was not expected; however, there appears to be interaction between anti-Spike and the TJP1 nanoplates. For this reason, further analysis was necessary to gain a better understanding of the possible causes of binding; thus, dynamic light scattering (DLS) measurements were carried out for all treatments following the addition of antibodies.

### 2.6. Dinamic Light Scattering (DLS) Size Measurements

**Figure 5:**
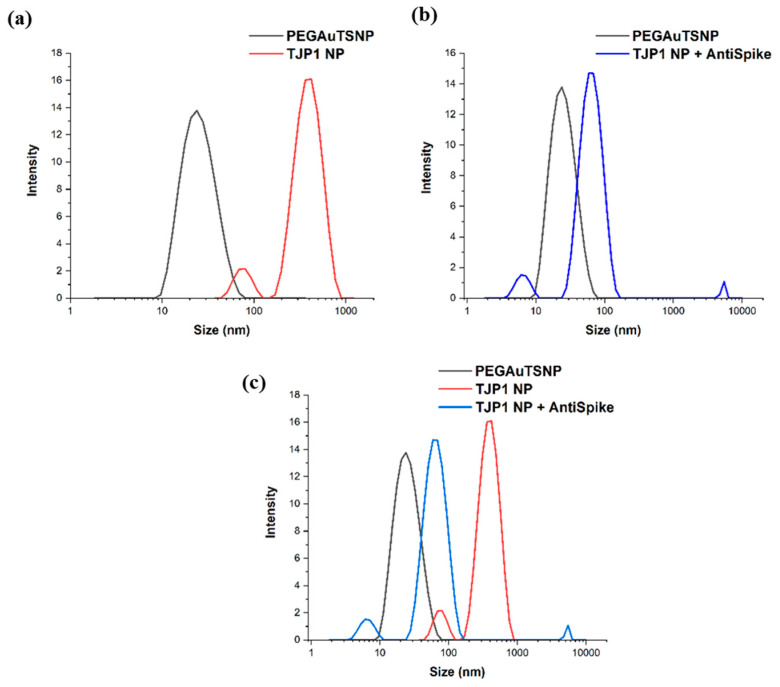
DLS size measurements of (**a**) PEGAuTSNPs and TJP1-PEGAuTSNPs, (**b**) TJP1-PEGAuTSNPs + anti-Spike, (**c**) superimposed graphs of (**a**,**b**).

Table S1 shows the DLS size measurements for all treatments before and after exposure to anti-Spike. In most cases, it can be observed that there was an increase in the average size of the particles after the apparent binding of the antibody. According to Figure 5, DLS showed a size measurement of 395.9 nm for TJP1-PEGAuTSNPs, which only corresponds to an 8 nm shift in the LSPR spectrum, as stated in Table 6 (from 589 nm to 597 nm). Upon anti-Spike addition, there was a reduction in size to 53.53 nm; however, there was an LSPR recording of a 17 nm shift, which could be attributed to the replacement of TJP1 by anti-Spike on the PEGAuTSNPs’ surfaces.

### 2.7. Spike–Anti-Spike Binding PEGAuTSNP-Based Immunoassay within Horse Serum

**Figure 6:**
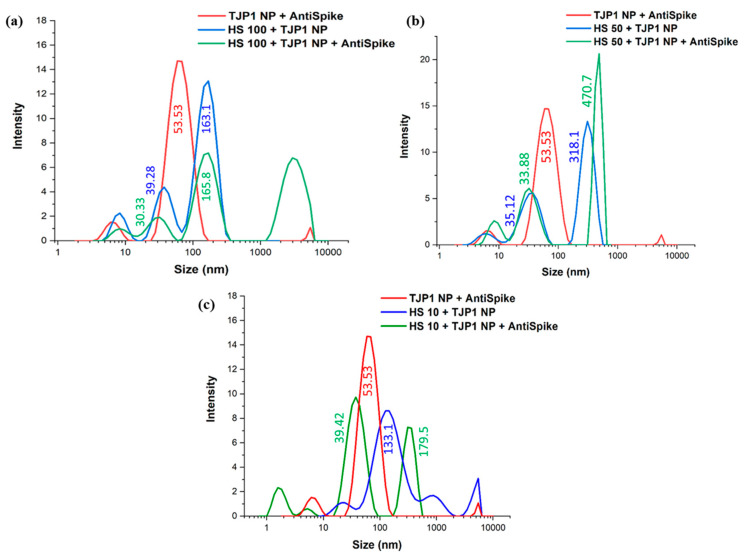
DLS measurements for TJP1-NP + anti-Spike and TJP1-NP within (**a**) 100%, (**b**) 50% and (**c**) 10% HS before and after addition of anti-Spike.

For the system within horse serum, a more detailed analysis of the results revealed that upon the addition of anti-Spike, there was an increase in size only for a small population of particles. In the case of 100% HS (HS100) with TJP1 nanoplates (Figure 6a), an average size of 142.1 nm was recorded with a Polydispersity Index (PI) of 0.33; after the sample interacted with the antibody, there was an increase in size to an average of 172.5 nm and PI of 0.78.

When the results are compared to TJP1 NP + anti-Spike (red plot, 53.53 nm in size), resemblance can be observed with the HS100 + TJP1 NP plot (blue), where a size of 39.28 nm was recorded. When the system is analyzed as a whole, namely HS100 + TJP1 NP + anti-Spike, a peak of 163.1 nm (blue plot) can be related to the 165.8 nm peak from the green-colored plot, suggesting the TJP1-coated nanoparticles did not bind to anti-Spike and kept their size; nonetheless, a fourth peak in the green plot above the 1000 nm region could be attributed to the anti-Spike binding and networking with components from the HS.

Fifty percent diluted horse serum (HS50) behaved in a similar manner, where an increase in the average size was observed with the presence of anti-Spike in the sample. The 50% HS with TJP1 nanoplates sample (Figure 6b) with an average size of 211.2 nm and a PI of 0.4 showed an increase to a size of 755 nm and PI of 0.69 as a consequence of higher polydispersity. Similar to HS100, relation can be observed between the plots, where TJP1 NP + anti-Spike (red plot) and HS50 + TJP1 NP (blue plot) can be identified within the HS50 + TJP1 NP + anti-Spike sample (green plot), where it can be observed that a fraction of the HS50 + TJP1 nanoplates interacted with the antibody, resulting in a higher intensity peak of 470.7 nm. 

In line with the previous HS concentrations, TJP1 NP + anti-Spike and HS10 + TJP1 NP can be identified within the most diluted sample (Figure 6c). Similar to the other HS samples, the plot suggests the TJP1-coated nanoplates did not bind to anti-Spike, but the antibody created networks with serum contents, resulting in a peak in the highest region among all samples (179.5 nm). Moreover, it can be observed that, comparable with the above results, the size of the HS10 + TJP1 NP sample was 133.1 nm (blue plot), and upon anti-Spike addition, a fraction of the sample reduced in size to 39.42 nm (green), validating the hypothesis that the antibody breaks the TJP1 network, causing the size reduction.

## 3. Discussion

Fibronectin (Fn), a critical ECM protein whose functions are governed by its conformational activity, is receiving increasing attention due to its participation in various phases of tumor proliferation and other complications of medical importance such as Fn glomerulopathy [25,26,27]. In this study, the suitability of PEGAuTSNPs for use as platforms for IAs was successfully demonstrated. The effective protein volumes to functionalize the nanoparticles and avoid potential non-specific binding were determined. Ab-Ag binding was detected within a complex environment where native Fn from the extracellular matrix was successfully detected by using anti-Fn antibody-coated nanoplates. 

Spike-PEGAuTSNPs’ detection limit was determined as a linear range of anti-Spike concentration between 0.01 and 0.1 mg/mL. In a comparative study of [28], detection limit ranges of rapid antigen tests were recorded between 9.8 and 78.6 ng/mL. This opens an area of opportunity to improve the proposed technique detection limits. 

The results of the functionalization of PEGAuTSNPs’ λ_max_ with anti-Fn (a shift of 65 nm) indicated the successful coating of the nanoplates with the antibody. However, when the functionalized nanoplates were exposed to different concentrations of cell-isolated ECM and analyzed, they showed a shifting profile, different than expected, with longer shifts recorded for the less concentrated ECM samples. One of the reasons could be attributed to the clumping of ECM proteins as a result of the cell scraping performed in the isolation protocol, impeding Fn binding sites to be sufficiently exposed to bind to its corresponding antibody; once the sample is diluted, the proteins disaggregate and Fn is exposed. According to Sediq et al., one of the reasons for protein aggregation is the induced mechanical stress during the friction of two solid surfaces, which, in this case, were the cell scraper and the flask containing the cell culture [29]. In future testing, ECM samples can be briefly homogenized to make the proteins scatter and aid in binding site recognition for the antigen of interest. Our results also implied that the TJP1 antibody did not recognize any epitope from MC3T3-E1 ECM proteins [30].

When it comes to the determination of optimal volumes for Spike protein functionalization, the results indicated the binding of the antibody only to the Spike protein available on the now fully functionalized nanoplates surface. The latter can be confirmed through the analysis of the Δλ, where it can be observed that between the concentrations of 5 µg and 10 µg of Spike in PEGAuTSNPs, the shift difference is constantly between 6 nm and 7 nm, indicating the maximum possible binding of the anti-Spike to Spike was reached. After the Spike–anti-Spike system specificity analysis, a reduced shift of the TJP1 nanoplates (25 nm) compared to the Spike nanoplates (65 nm) was observed, suggesting weak attachment of the TJP1 protein to the PEGAuTSNPs’ surfaces. This can also be inferred when evident binding between the antibody and its Spike counterpart (7 nm) was observed, but there was unexpected interaction between anti-Spike and TJP1, where there was almost no shifting (1 nm). This behavior could indicate no recognition of the antibody, which would be expected, or a stronger affinity of the anti-Spike for the nanoplates, causing a replacement on the nanoplates’ surface. 

The binding of Spike to anti-Spike was analyzed in the presence of HS to simulate a complex environment. As mentioned in the Results section, after TJP1-PEGAuTSNPs were exposed to anti-Spike, a minimal shift was recorded, similar to previous experiments; however, shifts of 8 nm, 3 nm and 2 nm were recorded for TJP1-NP upon addition of anti-Spike in the presence of 100%, 50% and 10% HS, respectively. These unanticipated positive shifts in relation to TJP1 could be associated with the non-specific binding of the HS components to the added anti-Spike. According to [31], HS is a non-standardized component of blood with varying concentrations of several proteins, albumins and globulins, among other components; this could interrupt, obstruct or, in this case, interfere with the predicted experiment behavior.

These results were further analyzed with DLS measurements, where several nanoparticle populations of varying sizes were identified in the samples, explaining the outcomes of the experiment. DLS size measurements showed the increase in the average size of the particles after the apparent binding of the antibody in most cases; nonetheless, three peaks could be observed in all samples containing HS even before exposure to anti-Spike, possibly indicating the coexistence of nanoparticles of different sizes within the same sample or agglomeration of HS proteins [32]. A similar phenomenon was observed in the study of Schintke and Frau (2020), where the interaction between nanoparticles and mouse serum sample showed poor signal stability in DLS measurements as the unfiltered serum sample contained large agglomerates that affected the particle size, causing several peaks [33]. The use of TJP1 as a control showed interesting performance within HS samples, and thus, it could be concluded that protein–protein networks were formed which were then broken up by the added anti-Spike antibody due to its stronger affinity to the nanoplates.

**Figure 7:**
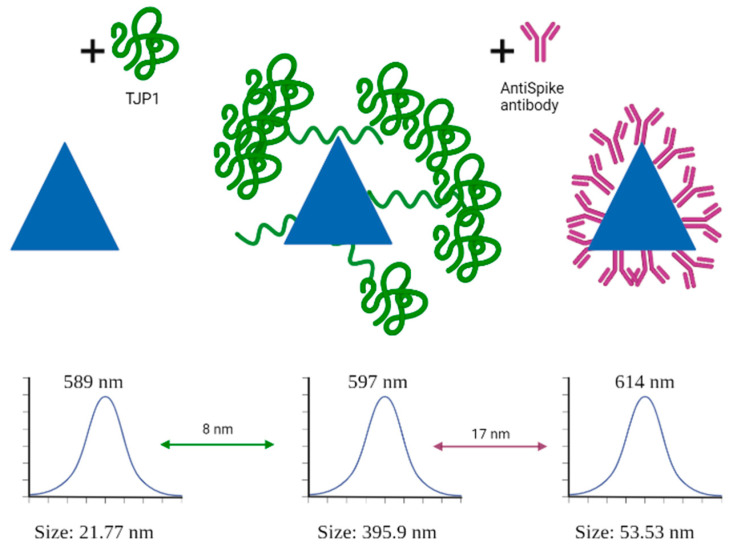
Illustration of protein replacement due to stronger attachment of anti-Spike to PEGAuTSNPs.

As mentioned before, anti-Spike seemed to show stronger affinity for the nanoplates, causing TJP1 to be replaced by the antibody on the nanoplates’ surface (Figure 7). DLS measurements for TJP1-PEGAuTSNPs could be attributed to TJP1 causing networking by itself. Since networking is not a close reaction to the PEGAuTSNP surface, it does not reflect in a large shift (8 nm) [25]. Nonetheless, upon anti-Spike addition, there was a reduction in size, possibly indicating the breakage of the TJP1 network on the nanoplates. The anti-Spike antibody seemed to show a stronger affinity for the nanoplates, causing the TJP1 protein to be replaced by the antibody on the nanoplates’ surface, reflected in a redshift. Nonetheless, the formed TJP1 network may be broken up, causing less protein molecules on the surface of the nanoplate, resulting in a DLS size reduction [25].

In future research, alternative complex environments and controls to validate the results will be assessed, and additional steps such as the filtering of bigger molecules within the samples to minimize non-specific binding and polydispersity will be taken. 

## 4. Materials and Methods

### 4.1. Materials

Fibronectin bovine plasma (1 mg/mL) (F4759-1G), Horse Serum donor herd (H1270-500ML) and anti-FN antibody (1 mg/mL) (AV41490-100UL) were obtained from Sigma Aldrich (St. Louis, MO, USA). The MC3T3-E1 pre-osteoblast cell line (CRL-2593) was obtained from ATCC, and Gibco Alpha Minimum Essential Medium (41061029), Invitrogen SARS-CoV-2 Spike Protein (RBD) Polyclonal Antibody (PA5-114451, 0.5–1 mg/mL) and Invitrogen SARS-CoV-2 Nucleocapsid/Spike Protein (RBD) Recombinant Protein (RP87706, 1 mg/mL) were obtained from Thermo Fisher (Waltham, MA, USA). Human TJP1 recombinant protein (APrEST83050, 0.5–1 mg/mL) and human-specific anti-TJP1 antibody (HPA001636, 0.5–1 mg/mL) were obtained from Atlas Antibodies (Stockholm, Sweden).

### 4.2. Anti-Fn-PEGAuTSNP Preparation

PEGAuTSNPs were synthesized through a seed-mediated approach where silver nitrate was reduced by sodium borohydride [9]. Subsequently, 1 mL of freshly synthesized nanoplates was incubated with 20 μg of anti-Fn antibody, with the concentration before nanoplate saturation being as described by Rodriguez Barroso et al. (2023). 

### 4.3. Extracellular Matrix Isolation Protocol

Extracellular matrix (ECM) isolation from cell culture was performed based on the protocol developed by [34] to study the interaction between native Fn from live cells and anti-Fn antibody. MC3T3-E1 pre-osteoblast cells were cultured and plated in a 6 well-plate at high density and placed in the incubator for 24 h until they were 95% confluent. Ammonium Hydroxide (NH_4_OH, 20 mM) was prepared with de-ionized water in an extractor hood. The culture plate was removed from the incubator, and the culture medium was removed from each well with a micropipette. Wells were gently rinsed with 1 mL of PBS without Ca^2+^/Mg^2+^, and the liquid containing PBS and remaining media were removed from the wells. Subsequently, 300 µL of 20 mM NH_4_OH was added to each well and rocked for a few seconds before washing each well with 1 mL of distilled water. The ammonium-hydroxide-solubilized material containing water, ammonium hydroxide and lysed cells was immediately removed with a micropipette. After that, the 6-well plate was tilted at an angle, and remaining ECM was collected from the bottom of the well by using a cell scrapper and placed in Eppendorf tubes. 

### 4.4. Detection of Native Fibronectin with Anti-Fn-PEGAuTSNP 

Three concentrations of MC3T3-E1 cells–isolated ECM (100%, 50% and 15%) were prepared with distilled water. ECM samples were further exposed to a concentration of 0.21 mg/mL of anti-Fn-PEGAuTSNPs (functionalized NPs before saturation point, as stated by Rodriguez Barroso et al. 2023) in a 1:1.5 ECM/anti-Fn-PEGAuTSNP ratio and placed on a 96-well plate to analyze interactions between native Fn and the antibody. Purified Fn samples in concentrations of 100% and 15% were used as positive controls for the binding of anti-Fn with Fn (1:1.5 Fn: Anti-Fn-PEGAuTSNP ratio). The 96-well plate was analyzed in the microplate reader. Results were compared to a second experiment where human-specific TJP1 antibody was used as a negative control for binding with the ECM isolated from cells. In order to detect changes in LSPR and λ_max_, absorbance spectra measurements were performed in a microplate reader adjusted from wavelengths 300–900 nm in 1 nm increments. 

### 4.5. Spike Protein Optimal Functionalization Volume Determination

PEGAuTSNPs were prepared as previously stated, and different amounts of spike protein were added to the nanoplates. Then, 1 mL of PEGAuTSNPs was placed in five Eppendorf tubes (5 mL total) and 1 µg, 3 µg, 5 µg, 7 µg and 10 µg of Spike protein were added to each tube. A sample of 100 µL was taken from each tube and placed on a 96-well plate. The plate was analyzed in an Ultraviolet–visible (UV-vis) spectrometer (Synergy HT BioTek microplate reader, Winooski, VT, USA) reader. After the first reading, 1 µg of anti-Spike antibody was added to each well, and the plate was analyzed again to observe changes in the LSPR. UV-Vis spectra measurements were adjusted from wavelengths 300–900 nm in 1 nm increments.

### 4.6. Nanoplate-Based Immunoassay with Changing Concentrations of Anti-Spike Antibody

After the suitable concentration of spike PEGAuTSNPs was determined, 4 µL spike/mL PEGAuTSNPs were used with different amounts of anti-Spike to determine the nanoparticle saturation point. A sample of 100 µL of Spike PEG-AuTSNPs was placed on a 96-well plate and analyzed in the plate reader. After that, 1 µg of anti-Spike was added to the well and the plate was analyzed again in a microplate reader with adjusted wavelengths from 300–900 nm in 1 nm increments. Following that, 2 µg, 4 µg, 6 µg, 8 µg and 10 µg were added to the same well, and the plate was analyzed between every addition. The final sum of anti-Spike in the well at the end of the experiment was 31 µg. Experiments were performed in the same well to avoid variations in the readings and to keep uniformity.

### 4.7. Spike–Anti-Spike Binding PEGAuTSNP-Based Immunoassay

Spike-PEGAuTSNPs were exposed to anti-Spike to test the nanoplate-based immunoassay system. Then, 100 µL of Spike-PEGAuTSNPs in a 4 μg Spike/mL PEGAuTSNP concentration and 100 µL of TJP1-PEGAuTSNPs (5 μg TJP1/mL PEGAuTSNP) were placed in a 96-well plate and analyzed on the plate reader. After the first reading, 4 µg of anti-Spike was added to both wells and the plate was analyzed again. The microplate reader was adjusted from wavelengths 300–900 nm in 1 nm increments for absorbance measurements.

### 4.8. Spike–Anti-Spike Binding PEGAuTSNP-Based Immunoassay within Horse Serum

The binding of the Spike–anti-Spike complex within horse serum as a high-noise environment was monitored using PEGAuTSNPs. Spike-PEGAuTSNPs were prepared with 4 µg of Spike in 1 mL of PEGAuTSNPs, and TJP1-PEGAuTSNPs were prepared in a similar concentration of 5 µg of TJP1 per 1 mL of PEGAuTSNPs. The latter was used as a negative control for binding with the Spike antibody. Three different concentrations of horse serum (100%, 50% and 10%) were prepared with distilled water to observe differences in the LSPR as a result of the high concentration of proteins within the serum. Samples were placed in a 96-well plate in a ratio of 1:1.67 horse serum/functionalized PEGAuTSNPs. The plate was analyzed in the plate reader. After the first analysis, 4 µg of anti-Spike was added to all wells, and the plate was analyzed again.

### 4.9. Dynamic Light Scattering (DLS) Size Measurements

Dynamic Light Scattering (DLS) is extensively used in research for the study of proteins, colloidal particles, and nanoparticles [33]. It studies the diffusion of molecules in solution through the diffusion coefficient and hydrodynamic radius, which are dependent on the analyzed molecules’ size and shape [35]. In this study, Zetasizer Pro (Malvern Panalytical Ltd., Malvern, UK) with DTS0012 polystyrene disposable cuvettes was used to determine the particle size of the samples.

All samples from Section 4.7 were diluted with ultrapure water in a 1:10 dilution. Then, 1 mL of the newly diluted sample was placed in the disposable cuvette and the cuvette lid was placed on top. Size measurements were repeated 3 times per sample at 25 °C, and the results were analyzed in ZS XPLORER software (https://www.malvernpanalytical.com/jp/support/product-support/software/zetasizer-ultra-pro-zs-xplorer-software-update-v1-00, accessed on 29 June 2023).

## 5. Conclusions

This study successfully demonstrated the potential of PEGAuTSNPs as platforms for This study successfully demonstrated the potential of PEGAuTSNPs as platforms for immunoassays, showcasing the effective protein volumes for functionalization and successful detection of native Fn using AntiFn antibody-coated nanoplates. When exposed to cell-isolated ECM samples, unexpected shifting profiles were observed, potentially attributed to protein clumping caused by the ECM-isolation protocol. Future testing could involve brief homogenization of ECM samples to aid in binding site recognition. On the other hand, successful binding of AntiSpike antibody to Spike protein on the fully functionalized nanoplates was demonstrated upon Spike protein optimal functionalization volume determination. Moreover, weak attachment of the control protein, TJP1, to the nanoplates was observed. The detection limit of the Spike-PEGAuTSNP ranged between 0.01–0.1 mg/mL, while the detection limits reported for rapid antigen tests range between 9.8–78.6 ng/mL. This opens an area of opportunity to improve the detection limits of the Spike-PEGAuTSNP system. In the presence of complex environments such as HS, unanticipated positive shifts in TJP1 nanoplates were recorded, possibly due to non-specific binding of HS components. DLS measurements revealed the coexistence of nanoparticle populations of varying sizes and protein-protein networks formation within horse serum samples. Future research should explore alternative complex environments, implement appropriate controls, and consider filtering larger molecules to minimize non-specific binding and polydispersity. Overall, this study provides valuable insights into the behavior of immunoassay platforms and highlights areas for further investigation and refinement.

## Figures and Tables

**Table 1:** λ_max_ of anti-Fn-PEGAuTSNP within different concentrations of ECM.

Treatment	λ_max_
PEGAuTSNP	638 nm
Anti-Fn-PEGAuTSNP	703 nm
100% ECM + Anti-Fn-PEGAuTSNP	707 nm
50% ECM + Anti-Fn-PEGAuTSNP	711 nm
15% ECM + Anti-Fn-PEGAuTSNP	715 nm
100% Fn + Anti-Fn-PEGAuTSNP	723 nm
15% Fn + Anti-Fn-PEGAuTSNP	714 nm

**Table 2:** λ_max_ of anti-TJP1-PEGAuTSNPs within different concentrations of ECM.

Treatment	λ_max_
PEGAuTSNP	638 nm
Anti-TJP1-PEGAuTSNP	675 nm
100% ECM + Anti-TJP1-PEGAuTSNP	677 nm
50% ECM + Anti-TJP1-PEGAuTSNP	677 nm
15% ECM + Anti-TJP1-PEGAuTSNP	671 nm
100% Fn + Anti-TJP1-PEGAuTSNP	678 nm

**Table 3:** λ_max_ recordings before and after the addition of anti-Spike.

Spike volume	1 µg	3 µg	5 µg	7 µg	10 µg
λ_max_ before anti-Spike (nm)	603	647	641	632	632
λ_max_ after anti-Spike (nm)	615	651	647	639	638
Δλ (nm)	12	4	6	7	6

**Table 4:** λ_max_ for Spike-PEGAuTSNPs and TJP1-PEGAuTSNPs before and after the addition of anti-Spike.

	λ_max_ Spike-PEGAuTSNP (nm)	λ_max_ TJP1-PEGAuTSNP (nm)
Before anti-Spike	639	600
After anti-Spike	646	601
Δλ	7	1

**Table 5:** λ_max_ for Spike-PEGAuTSNP and TJP1-PEGAuTSNP treatments within HS before and after exposure to anti-Spike.

	Spike NP	HS 100% + Spike	HS 50% + Spike	HS 10% + Spike	TJP1 NP	HS 100% + TJP1 NP	HS 50% + TJP1 NP	HS 10% + TJP1 NP
λ_max_ before anti-Spike (nm)	590	609	612	607	596	612	617	604
λ_max_ after anti-Spike (nm)	603	617	619	609	597	620	620	606
Δλ (nm)	13	8	7	2	1	8	3	2

**Table 6:** λ_max_ for TJP1-PEGAuTSNP treatments within HS before and after exposure to anti-Spike.

	PEG AuTSNP	Spike NP	TJP1 NP	HS 100% +TJP1 NP	HS 50% +TJP1 NP	HS 10% +TJP1 NP
λ_max_ before anti-Spike (nm)	589	604	597	628	627	618
λ_max_ after anti-Spike (nm)	614	619	614	633	632	620
Δλ	25	15	17	5	5	2

## Data Availability

zenodo.org, accessed on 15 June 2023.

## Supplementary Materials

The supporting information can be downloaded at: https://www.mdpi.com/article/10.3390/ijms241511974/s1.
