# Supplementary material for: Triangular Silver Nanoplates as a Bioanalytical Tool: Potential COVID-19 Detection
Source: Int J Mol Sci. 2023 Jul 26;24(15):11974. doi: 10.3390/ijms241511974 (PMC10418913; doi:10.3390/ijms241511974)
Supplement: Supplementary file 1 [file ijms-24-11974-s001.zip › ijms-2508206-supplementary.pdf]

Table S1. Size measurements of PEGAuTSNP, Spike-PEGAuTSNP and TJP1-PEGAuTSNP treatments within HS before and after exposure to AntiSpike.

<table>
<tr><th>Size measurement before AntiSpike</th><th>Size measurement after AntiSpike</th><th>Δλ</th></tr>
<tr><td>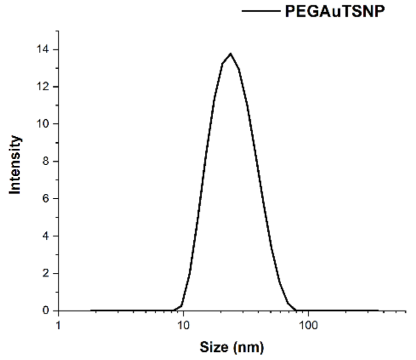

PEGAuTSNP (NP)
Size: 21.77 nm</td><td>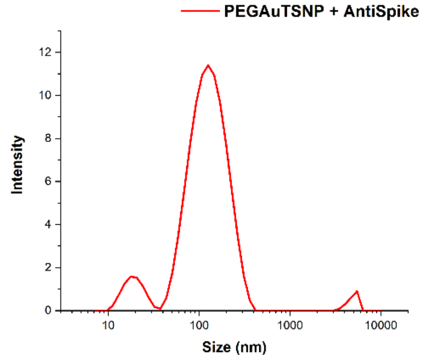

PEGAuTSNP + AntiSpike
Size: 90.34 nm</td><td>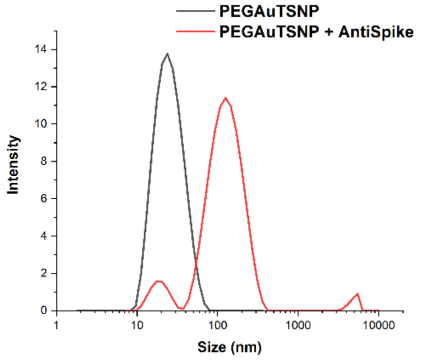

ΔSize: 68.57</td></tr>
<tr><td>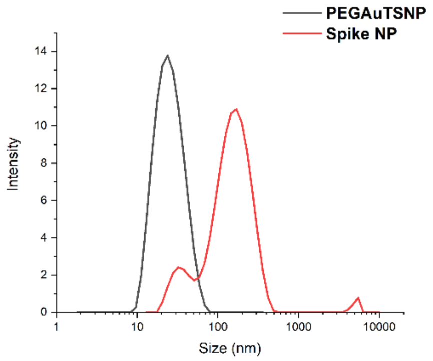

Spike NP
Size:107 nm</td><td>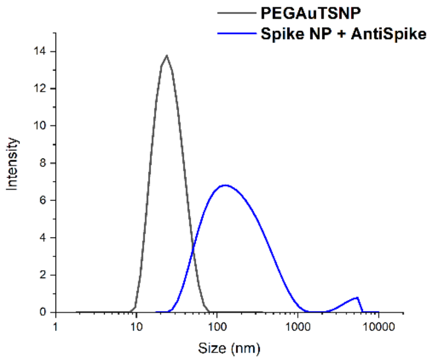

Spike NP + AntiSpike
Size: 119.7 nm</td><td>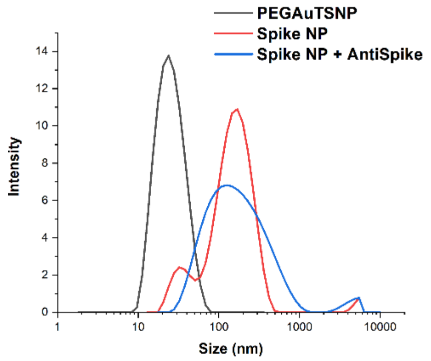

ΔSize: 12.7 nm</td></tr>
<tr><td>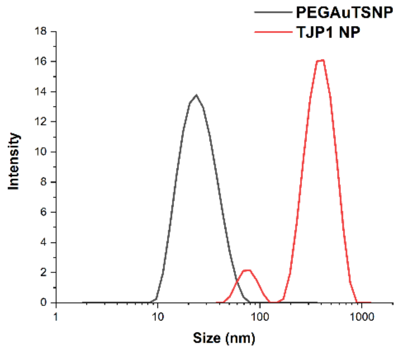

TJP1 NP
Size: 395.9</td><td>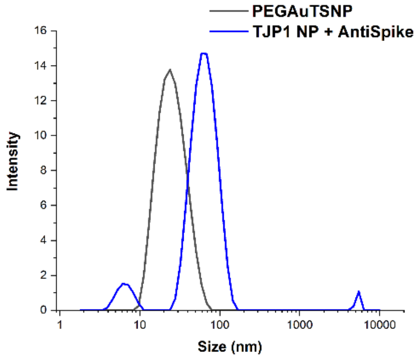

TJP1 NP + AntiSpike
Size: 53.53</td><td>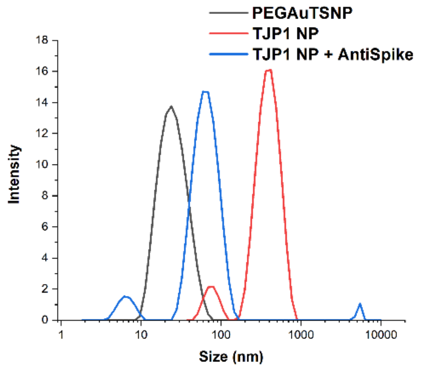

ΔSize: -342.37 nm</td></tr>
</table>

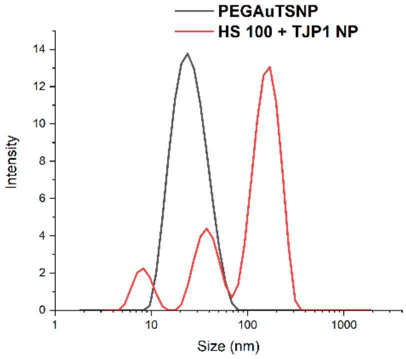

HS100% + TJP1 NP
Size: 142.1

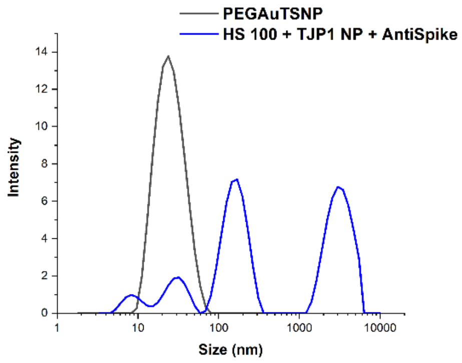

HS100% + TJP1 NP + AntiSpike
Size: 172.5 nm

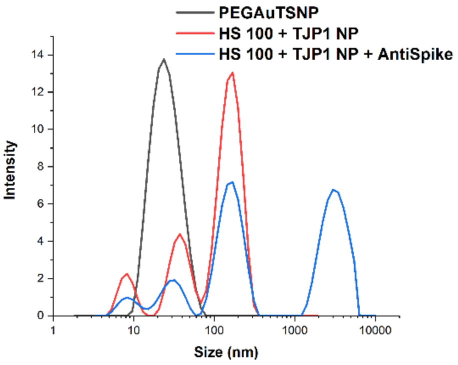

ΔSize: 30.4 nm

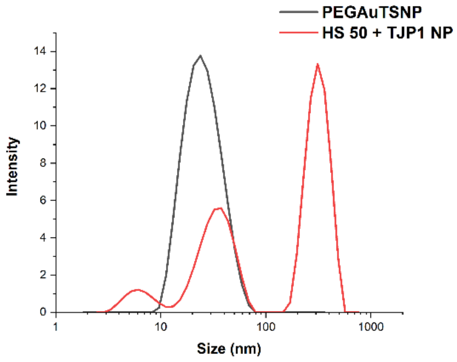

HS50% + TJP1 NP
Size: 211.2

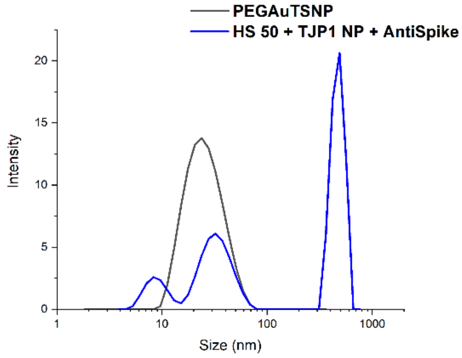

HS50% + TJP1 NP + AntiSpike
Size: 755 nm

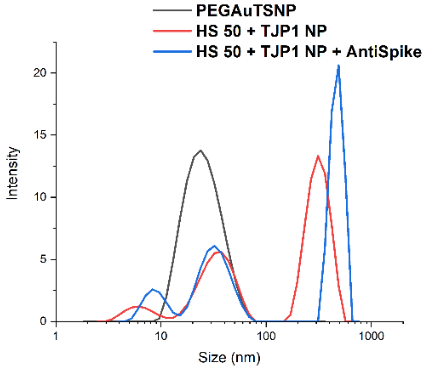

ΔSize: 543.8 nm

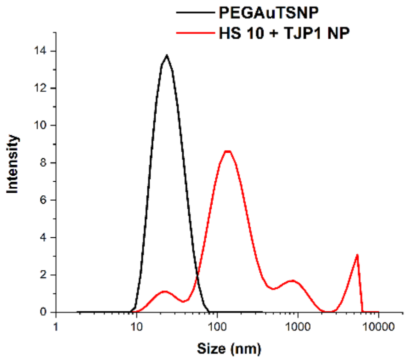

HS10% + TJP1 NP
Size:133.1

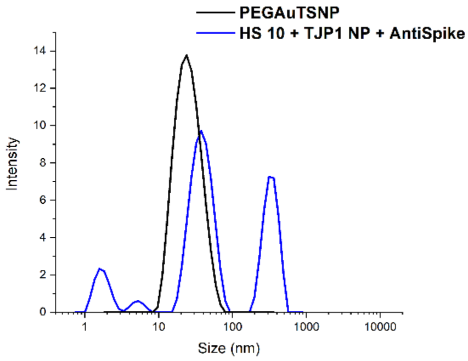

HS10% + TJP1 NP + AntiSpike
Size: 179.5 nm

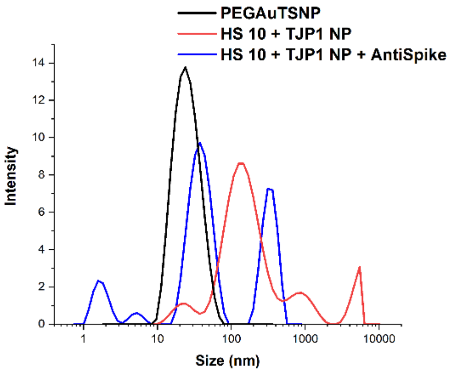

ΔSize: 46.4 nm
